# The speed of kill of fluralaner (Bravecto™) against *Ixodes ricinus* ticks on dogs

**DOI:** 10.1186/s13071-014-0525-3

**Published:** 2014-11-18

**Authors:** Christina Wengenmayer, Heike Williams, Eva Zschiesche, Andreas Moritz, Judith Langenstein, Rainer KA Roepke, Anja R Heckeroth

**Affiliations:** MSD Animal Health Innovation GmbH, Zur Propstei, 55270 Schwabenheim, Germany; Department of Veterinary Clinical Sciences, Clinical Pathophysiology and Clinical Pathology, Justus-Liebig-University Giessen, Giessen, Germany

**Keywords:** Bravecto™ chewable tablets, Fluralaner, Speed of kill, Dog, Tick, *Ixodes ricinus*, Tick-borne diseases, Efficacy

## Abstract

**Background:**

Pathogens that are transmitted by ticks to dogs, such as *Anaplasma phagocytophilum, Babesia* spp., *Borrelia burgdorferi* sensu latu, and *Ehrlichia canis,* are an increasing problem in the world. One method to prevent pathogen transmission to dogs is to kill the ticks before transmission occurs. Fluralaner (Bravecto™) is a novel isoxazoline insecticide and acaricide that provides long persistent antiparasitic activity following systemic administration. This study investigated the speed of kill of fluralaner against *Ixodes ricinus* ticks on dogs.

**Methods:**

A total of 48 dogs were randomized to 8 groups of 6 dogs and each dog was infested with 50 female and 10 male *I. ricinus* ticks. Two days later (day 0), 4 groups received a single treatment of 25 mg fluralaner/kg body weight as Bravecto™ chewable tablets; the dogs in the other 4 groups were left untreated. Separate control and treatment groups were paired at each time point (4, 8, 12, or 24 hours after treatment) for assessment of tick-killing efficacy. At 4, 8, and 12 weeks after treatment, all dogs were re-infested with 50 female *I. ricinus* ticks and subsequently assessed for live or dead ticks at either 4, 8, 12, or 24 hours after re-infestation. Efficacy was calculated for each assessment time point by comparison of the treatment group with the respective control group.

**Results:**

Tick-killing efficacy was 89.6% at 4 hours, 97.9% at 8 hours, and 100% at 12 and 24 hours after treatment. Eight hours after re-infestation, efficacy was 96.8%, 83.5%, and 45.8% at 4, 8, and 12 weeks after treatment, respectively. At least 98.1% tick-killing efficacy was demonstrated 12 and 24 hours after re-infestation over the entire 12 week study period.

**Conclusions:**

Fluralaner kills ticks rapidly after treatment at 4 hours, and over its entire 12-week period of efficacy, it achieves an almost complete killing effect within 12 hours after tick infestation. The rapid tick-killing effect together with the long duration of efficacy enables fluralaner to aid in the prevention of tick borne diseases.

## Background

Ticks are a common nuisance to humans and animals, as they not only feed on the blood of their host, but also carry numerous pathogens such as viruses, bacteria, and parasites. Through feeding, infected ticks can transmit pathogens to both human and domestic animal hosts, especially dogs [1,2]. Vector-borne diseases are a growing problem worldwide [3,4], due to increasing pet ownership, more owners traveling with their pets, and the ability of vector arthropods to establish themselves in new localities [5]. Ticks and tick-borne diseases are spreading worldwide and are no longer restricted to certain areas.

Prevention of tick-borne diseases can be achieved by avoiding tick habitats and by physical removal of ticks from infested dogs. A better approach is the use of treatments capable of repelling, or rapidly killing ticks prior to transmission, ideally with a long duration of efficacy [6-9].

Fluralaner (Bravecto™), a new ectoparasiticide that belongs to the novel isoxazoline compound class, is efficacious against *Ixodes ricinus, Ixodes scapularis, Dermacentor reticulatus, Dermacentor variabilis*, and *Rhipicephalus sanguineus*, i.e., against all tick species that potentially harbor the most relevant pathogens to humans and domestic animals, such as *Anaplasma phagocytophilum, Babesia* spp.*, Borrelia burgdorferi* sensu latu, and *Ehrlichia canis* [3].

Fluralaner’s mode of action is antagonism of ligand-gated chloride channels (both gamma-aminobutyric acid- (GABA) receptor and glutamate-receptor) that potently inhibit the arthropod nervous system [10], resulting in paralysis and death of fleas and ticks [11]. Fluralaner has a significantly high selectivity for arthropod versus mammalian neurons [10,12] and is well tolerated by dogs that are at least 8 weeks old, including MDR 1 (−/−) Collies [13,14].

In dogs, fluralaner has a long elimination half-life, a long mean residence time, a relatively high apparent volume of distribution, and a low clearance rate [15]. The long residence time of fluralaner in the dog’s plasma results in persistent killing activity on fleas and ticks for 12 weeks after a single oral treatment [16]. The efficacy of fluralaner depends on ticks attaching to the host’s skin, commencing feeding and thereby ingesting the active compound [11]. Sustained feeding increases risk of pathogen transmission from ticks to dogs, but transmission usually does not occur immediately after a tick attaches to a dog. Instead, an initial attachment and feeding period of at least 24 to 48 hours is required before transmission occurs in most tick-borne disease systems, a period in which reactivation of tick-borne pathogens takes place [2]. The time period between attachment and transmission allows a systemic ectoparasiticide such as fluralaner to take effect. If the infected ticks are killed within this time period, the transmission can most likely be prevented. Therefore, the speed of kill, defined as the time necessary to kill already attached ticks or to kill ticks after re-infestation, is an important factor in the prevention of tick-borne diseases.

Based on the rationale outlined above, 2 studies were conducted to evaluate the speed of kill of fluralaner by measuring the tick-killing efficacy at 4 and 8 hours (study 1) or 12 and 24 hours (study 2) after a single treatment, and at various time points after re-infestation of these dogs over the entire 12-week duration of efficacy.

In both studies, *I. ricinus* ticks were selected to infest the dogs, because this species is the vector for one of the most common tick-borne infections, Lyme borreliosis (caused by *B. burgdorferi*), in the temperate northern hemisphere [17].

## Methods

Both studies were in compliance with German animal welfare regulations, and ethical approval was obtained before start of the study by the “Landesuntersuchungsamt Rheinland-Pfalz”. The studies were conducted in accordance with the OECD Principles of Good Laboratory Practice (GLP) and the GLP Principles of the German Chemikaliengesetz (Chemicals Act). Both studies were conducted as blinded, randomized, negative controlled efficacy studies.

### Study design

The studies investigated the speed of kill of fluralaner against *I. ricinus* ticks on dogs. In total, 48 healthy adult Beagles (≤7 years) that had not been treated with any parasite control product for at least 12 weeks prior to starting this study were included. Prior to randomization, dogs were weighed (range 9.3–19.9 kg) and clinically examined. Participating dogs were previously infested with 80 fleas (*Ctenocephalides felis*) to demonstrate their susceptibility to parasite infestation and to prove the absence of ectocides. Ranking of the dogs was performed by descending flea counts (between 63 – 80 fleas/dog) and dogs were randomly allocated to 8 study groups (4 treatment and 4 corresponding control groups) of 6 dogs each using a computer generated randomization list.

All dogs were kept indoors. During periods without parasite infestation, dogs were group-housed within their corresponding study group, while during periods of parasite infestations, all dogs were housed individually. Temperature in the dog housing facility ranged between 17 – 22°C and the relative humidity between 40 – 90%. Dogs were fed a standard commercially available dry dog food once daily, and drinking water was provided *ad libitum*. General health observations were performed once daily throughout the study.

### Treatment

On day 0 (i.e., day of treatment), dogs in the 4 treatment groups received fluralaner chewable tablets based on the dog’s individual body weight to achieve a minimum dose of 25 mg fluralaner/kg body weight. The fluralaner chewable tablets were administered by placement in the back of the oral cavity over the tongue to initiate swallowing. Dogs received half of their daily food ration within 20 minutes prior to treatment and the balance immediately thereafter. Each dog was continuously observed for 1 hour after administration to assess whether chewable tablets were vomited or spit out, which did not occur. Dogs in the 4 control groups were left untreated.

### Tick infestations and assessments

The *I. ricinus* ticks used in the studies were reared in a laboratory for at least 2 generations since their introduction from the wild (Europe). Tick infestations were conducted on medetomidin sedated dogs on days -2, 28 (4 weeks), 56 (8 weeks) and 84 (12 weeks). At each infestation time point, every dog was infested with 50 female unfed adult ticks applied directly to their fur along the back, lateral side, and head. Approximately 10 male ticks were additionally applied to give *Ixodes* females best conditions for attachment. Tick-infested dogs were held in individual pens until parasite removal. The tick burden (male and female ticks) of all dogs in each treatment and control group pairing were assessed at either 4 (±0.75), 8 (±0.75), 12 (±1.5), or 24 (±1.5) hours after treatment (week 0) or after re-infestation (weeks 4, 8, and 12). The entire body of each dog was examined and ticks were carefully removed using forceps. Ticks were classified as dead or alive, attached or unattached, and counted. Personnel conducting tick classification and tick counts were blinded to the treatment status of each dog.

### Statistical analysis

The statistical analysis was performed using the software package SAS® (SAS Institute Inc., Cary, NC, USA, release 9.2). The individual animal was the statistical unit in all statistical calculations. The percentage of tick efficacy was calculated for each treated group and assessment time point according to Abbott’s formula:

Efficacy (%) = 100 x (M_C_ – M_T_) / M_C_, where Mc is the geometric mean of live ticks on control group dogs and M_T_ is the geometric mean of live ticks on treatment group dogs. In case of zero counts, the geometric mean of ticks (x _g_) was calculated as follows:

$$ {\mathrm{x}}_{\mathrm{g}}={\left({\displaystyle \prod_{\mathrm{i}=1}^{\mathrm{n}}\left({\mathrm{x}}_{\mathrm{i}}+1\right)}\right)}^{\frac{1}{\mathrm{n}}}-1 $$

where n is the number of animals, i the index and x_i_ is the number of ticks on the i-th animal.

Significance of differences was assessed between the log-counts of the live ticks of each treated group in comparison with the log-counts of the respective untreated control group for each assessment time point. The treated and control groups were compared using a linear mixed model that includes treatment group as a fixed effect and block as a random effect. The two-sided level of significance for the F-tests of the model effects was set to α = 0.05.

## Results

No treatment-related adverse events were observed in any of the 24 fluralaner-treated dogs during the 12-week post-treatment observation period. The mean tick counts and the detailed efficacy results are shown in Table 1. Tick-killing efficacy was 89.6% at 4 hours, 97.9% at 8 hours, and 100% at 12 and 24 hours after treatment. Eight hours after re-infestation, efficacy was 96.8%, 83.5%, and 45.8% at 4, 8, and 12 weeks after treatment, respectively. At least 98.1% tick-killing efficacy was demonstrated 12 and 24 hours after re-infestation over the entire 12 week study period.

**Table 1:** **Mean tick counts and tick-killing efficacy (%) after oral administration of fluralaner to dogs**

**Assessment time point** ^**a**^		**4 hours**	**8 hours**	**12 hours**	**24 hours**
**Week 0**	Mean tick counts^b^ (control/treated) [n]	33.0/3.4	30.6/0.6	28.1/0	15.8/0
	Efficacy [%]	89.6^#^	97.9^#^	100^#^	100^#^
**Week 4**	Mean tick counts^b^ (control/treated) [n]	36.5/24.4	40.5/1.3	36.7/0.1	30.6/0
	Efficacy [%]	33.2*	96.8^#^	99.7^#^	100^#^
**Week 8**	Mean tick counts^b^ (control/treated) [n]	38.6/31.8	31.2/5.2	32.0/0.3	29.8/0.1
	Efficacy [%]	17.5	83.5^#^	99.2^#^	99.6^#^
**Week 12**	Mean tick counts^b^ (control/treated) [n]	37.4/34.5	30.0/16.2	34.3/0.6	32.7/0.6
	Efficacy [%]	7.8	45.8^§^	98.3^#^	98.1^#^

^a^Assessment for ticks in hours after treatment or re-infestation.

^b^Geometric mean.

*Log-counts of live ticks from the treated group were significantly different (p < 0.03) from log-counts of the respective untreated control group.

^**§**^Log counts of live ticks from the treated group were significantly different (p < 0.004) from log-counts of the respective untreated control group.

^**#**^Log counts of live ticks from the treated group were significantly different (p < 0.0001) from log-counts of the respective untreated control group.

Tick counts 4, 8, 12 or 24 hours after treatment were significantly lower (p < 0.0001) in fluralaner treated dogs compared with tick counts from untreated control dogs. Tick counts assessed 8, 12 or 24 hours after re-infestation were also significantly lower (at all time points p < 0.0001; 8 hours following re-infestation in week 12: p < 0.004) in fluralaner treated dogs compared with untreated control dogs. Significant lower tick counts were also assessed 4 hours after re-infestation in week 4 (p < 0.03).

## Discussion

Fluralaner (Bravecto™) is the first orally administered ectoparasiticide to exhibit an extended period of efficacy against ticks. The present study clearly demonstrate that fluralaner rapidly kills ticks with increasing efficacy over time, reaching full efficacy at 12 hours for the entire 12-week duration of efficacy.

The systemic mode of action of fluralaner necessitates some uptake of the host’s body fluid by the ticks. The fast killing effect limits this uptake to a short period of time. The transmission of tick-borne disease pathogens to the host does not usually occur immediately after tick attachment, but after sustained feeding.

Pathogens are activated directly in the salivary glands prior to transmission or require a reactivation period to replicate and migrate to the salivary glands [2]. This reactivation period starts upon attachment to the host and the transmission takes place when the tick regurgitates excess fluid into the bite wound [2,18].

Several studies have been conducted to assess the time necessary for pathogen transmission [9,19-21]. Infected ticks were allowed to feed on host animals for defined time periods. After removal of the ticks, the host was checked for the absence or presence of the pathogen, or a specific immune reaction. If the data indicated that the host was in contact with the pathogen, the time the tick was on the host was considered sufficient for pathogen transmission.

For example, the transmission of *B. burgdorferi* to mice by *Ixodes* nymphs did not occur prior to 24 hours after attachment: 0 out of 18 mice became infected after exposure to *B. burgdorferi-*infected *I. ricinus* nymphs for 24 or 48 hours, and none of 58 mice were infected after exposure to *B. burgdorferi-*infected *I. scapularis* nymphs for 24 hours [20,21]. For *Babesia canis*, transmission from infected *D. reticulatus* ticks to dogs did not occur prior to 48 hours [19]. Furthermore, Prullage *et al.,* concluded that the transmission rates of *B. burgdorferi, A. phagocytophilum*, and *B. microti* drop significantly if ticks are prevented from feeding for longer than 24 hours [22].

Once transmission is possible, the probability of pathogen transmission increases significantly and continues to increase with the duration of the blood meal [21]. Comparing these transmission times with the results of our study results, we conclude that fluralaner kills ticks before transmission of *Borrelia* and *Babesia* pathogens commences.

The *A. phagocytophilum* and *E. canis* pathogens are harbored in the midgut, but can also be found in salivary glands, which makes an earlier transmission possible. For example, the transmission of *A. phagocytophilum* from *I. scapularis* nymphs to mice occurs within the first 24 hours [20] and the transmission of *E. canis* occurs within 3–6 hours [9]. Thus, the risk of transmission of such pathogens cannot be completely excluded when considering the speed of kill of fluralaner and the reported transmission times from laboratory investigations. However, these studies were conducted under laboratory conditions; all existing variables of the vector:host natural state (e.g., vector infection rate and pressure, interrupted tick feeding, variations in the age, breed, and immune status of the host, and presence of other reservoir hosts) cannot be replicated in a laboratory. More importantly, in several studies conducted in dogs the use of commercial acaricidal products protected from an *A. phagocytophilum* or *E. canis* infection [8,23-27]. These products are described as having a slower speed of kill (i.e., time needed after treatment/re-infestation to kill >90% of attached ticks) compared with fluralaner [23,28-31]. Thus, fluralaner is likely to have at least the same ability to prevent *A. phagocytophilum* or *E. canis* infection in dogs.

The systemic mode of action of fluralaner adds some more advantages for the user over substances that are applied topically or by a collar and remain on the surface of a dog. A decrease of tick efficacy due to the loss of a collar or by swimming is possible after the use of respective products. Furthermore, the fact that the ticks are killed after feeding on a fluralaner treated dog also reduces the possibility of another dog getting infected as it can happen with solely repellent active substances.

Additionally to its rapid speed of kill and the systemic mode of action, the long duration of efficacy of fluralaner benefits owners by permitting fewer treatments over time than other commercially available monthly acaricidal products. Frequent and consistent use of ectoparasiticides is a major tool in tick-borne disease prevention. However, owner compliance is often poor [32,33], resulting in times when the dog is not protected [34], i.e. when monthly treatments are not re-administered in the recommended treatment interval. This puts not only the dog’s health at risk, but poses a risk to other dogs and humans, because an unprotected dog is a potential pathogen reservoir. Therefore, an ectoparasiticide with a rapid speed of kill and long duration of efficacy, such as fluralaner (Bravecto™), helps in the prevention and control of tick-borne diseases.

## Conclusions

Fluralaner kills ticks rapidly after treatment at 4 hours, and over its entire 12-week period of efficacy, it achieves an almost complete killing effect within 12 hours after tick infestation. The systemic mode of action of fluralaner is of advantage as it thus overcomes the decrease of efficacy due to the loss of a collar or swimming of the dog. The 12-week duration of efficacy is an additional benefit as it overcomes poor owner compliance regarding re-treatment. The rapid tick-killing effect together with the long duration of efficacy enables fluralaner to aid in the prevention of tick borne diseases.
